# A Cross-Sectional Survey of Risks Associated With Smoking Frequency and Cessation Practices Among Active Inpatient Smokers Admitted to a Tertiary Care Hospital in Jeddah, Saudi Arabia

**DOI:** 10.7759/cureus.101690

**Published:** 2026-01-16

**Authors:** Muhammad Sohaib Ejaz Khan, Abdulrahman Bazi, Abdulmalek Aboulkhair, Samirah Alghamdi, Bassam Alsaeedi, Basel Alghamdi

**Affiliations:** 1 Department of Internal Medicine, King Fahad Armed Forces Hospital, Jeddah, SAU

**Keywords:** cessation practices, inpatients, risk awareness, saudi arabia, smoking behavior

## Abstract

Introduction and aim: Tobacco smoking remains a major public health concern worldwide and is associated with multiple preventable diseases, including cancer, cardiovascular disorders, and chronic respiratory conditions. Despite sustained global and regional tobacco control efforts, smoking prevalence remains high in Saudi Arabia. This study aimed to assess smoking frequency, awareness of smoking-related health risks, and cessation practices among hospitalized active smokers admitted to a tertiary care hospital in Jeddah.

Methodology: A cross-sectional descriptive survey was conducted between October 2024 and April 2025 among 250 actively smoking inpatients admitted to the Department of Medicine at King Fahad Armed Forces Hospital, Jeddah. Data were collected using a validated, Arabic-language translated electronic questionnaire that captured socio-demographic characteristics, smoking behaviors, awareness of health risks, and cessation practices. Statistical analysis was performed using SPSS version 24 (Armonk, NY: IBM Corp.), employing descriptive statistics and chi-square tests, with statistical significance set at p≤0.05.

Results: The mean duration of smoking was 12.84±6.42 years, and the mean age at smoking initiation was 18.62±3.75 years. Cigarette smoking was the most prevalent form of tobacco use, reported by 191 participants (76.4%), followed by shisha use in 34 (13.6%) and dual use in 25 (10.0%). Awareness of smoking-related health risks was highest for lung cancer (212; 84.8%), whereas awareness of adverse pregnancy outcomes was comparatively low (94; 37.6%). More than half of the participants (139; 55.6%) reported at least one previous quit attempt; however, sustained abstinence for more than six months was achieved by only 34 (13.6%). Stratified analysis demonstrated significant associations between smoking behaviors and gender (p=0.021), age (p=0.034), marital status (p=0.013), and education level (p=0.033).

Conclusion: Hospital admission represents a critical window of opportunity to implement structured smoking cessation interventions, address gaps in risk awareness, and reduce socio-demographic disparities in smoking behaviors among active inpatient smokers.

## Introduction

Tobacco smoking is currently among the major preventable morbidity and mortality causes across the globe [1]. The use of cigarettes and other tobacco products leads to a significant proportion of cardiovascular disease, chronic respiratory disease, various cancers (including lung cancer), and many other adverse perinatal outcomes; tobacco around the world causes millions of deaths each year and has significant health and economic impacts on health systems [2,3]. Recent reports around the world have estimated that the use of tobacco still accounts for over 7-8 million deaths annually and still plays a significant role in the number of years of disability-adjusted life in parts of the world [4,5].

The trends of tobacco consumption are shifting as follows: despite the weakening prevalence in most high-income countries over the last 20 years, the shift towards new nicotine products (e-cigarettes, heated tobacco products) has complicated cessation interventions and exposed adolescents and young adults to higher levels of nicotine in a range of environments [6,7]. The world situation in tobacco control is thus confronted with the twofold issue of cutting down on traditional cigarette smoking and dealing with the fast adoption of the substitute nicotine delivery products [8].

Tobacco use is a significant issue in the Kingdom of Saudi Arabia (KSA) that has strong gender and regional disparities [9]. Adult tobacco-use prevalence estimates have been reported on national and regional surveys and differ by study and year, with recent analysis of the percentage of adult tobacco use ranging approximately in the low through high-teens overall, and in the high-teens percentile prevalence of tobacco use being significantly higher in men compared to women. The threat at the population level is also increased by exposure to second-hand tobacco smoke and the growing popularity of waterpipe and e-cigarette products. Such tendencies have been reported in several recent Saudi studies and population-health reports [10,11].

Hospitals and tertiary care centers are in a strategic position in the processes of tobacco control since the patients admitted often have tobacco-related diseases (or comorbid conditions further aggravated by smoking) and an admission provides an educational opportunity to initiate cessation programs. Although this is available, Saudi and regional literature reveal that screening of tobacco consumption may be inconsistent in clinical practice, and access to, or use of, cessation counselling and pharmacotherapy may be inconsistent. The inpatient smoking frequency, its associated risks, and actual cessation behavior in tertiary hospitals are thus critical to understanding how to tailor realistic hospital-based cessation pathways [12,13].

Local evidence of Jeddah and the Makkah region indicates that the nicotine dependence burden remains among the population seeking healthcare services, and the need is evident, but there is little data on a hospital level, especially on those that correlate smoking frequency (daily vs. intermittent, cigarettes vs. waterpipe/e-cigarette), clinical risk profiles, and actual cessation activities provided to patients during hospital admission. There are missing links in the routine evaluation of smokers and follow-ups, which have been reported in regional cross-sectional surveys and audits of the clinics, and highlight gaps in intervention opportunities [14].

Jeddah’s tertiary care hospitals serve a large and diverse patient population, with a substantial proportion presenting with smoking-related or smoking-exacerbated conditions. A hospital-wide inpatient assessment of smoking frequency, integrating evaluation of clinical risks (cardiovascular, respiratory, oncologic, and metabolic) and documentation of cessation interventions provided or utilized during hospital admission, offers actionable evidence to strengthen inpatient tobacco-cessation pathways. Such evidence can inform targeted clinician training, optimize allocation of resources to cessation services (including behavioral counseling and pharmacotherapy), and support policy initiatives to embed routine smoking assessment within standard admission procedures. In the context of the expanding market of nicotine products and prevailing local tobacco-use patterns, up-to-date hospital-based data from Jeddah are both timely and necessary. Accordingly, this study aimed to determine smoking frequency-associated clinical risks and existing cessation practices among active smokers admitted to a tertiary care hospital in Jeddah, Saudi Arabia.

## Materials and methods

Study design, setting, population, and duration

This was a cross-sectional descriptive survey conducted among patients admitted to the Department of Medicine at King Fahad Armed Forces Hospital (KFAFH), Jeddah, Saudi Arabia. The study population included actively smoking patients aged 14 years or older of both genders who were admitted during the study period. The survey was carried out over a six-month period, from October 1, 2024, to April 30, 2025, and data collection was completed prior to analysis.

Ethical approval

Ethical approval was obtained from the Research Ethics Committee of King Fahad Armed Forces Hospital (KFAFH), Jeddah (approval no: REC721, dated September 9, 2024). The study was conducted in accordance with institutional ethical guidelines and the Declaration of Helsinki. For participants aged 14-17 years, assent from the participant and consent from a parent or legal guardian were obtained prior to inclusion in the study.

Inclusion and exclusion criteria

Inclusion criteria comprised patients aged 14 years or older who were current (active) smokers and willing to participate in the study. For participants aged 14-17 years, assent from the participant and consent from a parent or legal guardian were obtained in accordance with institutional ethical guidelines. Exclusion criteria included patients unwilling to participate, those with cognitive impairment or an inability to complete the survey due to a medical condition, patients younger than 14 years, non-smokers, ex-smokers, and individuals not admitted during the study period.

Sample size calculation

The sample size was calculated using the Raosoft online calculator (Seattle, WA: Raosoft, Inc.) with a 90% confidence interval, 10% margin of error, and 50% response distribution, an approach commonly used for descriptive cross-sectional hospital-based surveys, yielding a required sample of 250 participants [15].

Data collection procedure

Data collection was initiated after obtaining ethical approval from the Research Ethics Committee of King Fahad Armed Forces Hospital (KFAFH). Eligible inpatients admitted to the medical wards were identified and approached by the research team. Each patient received a clear explanation of the study purpose, procedures, and their rights as participants, including confidentiality and the right to withdraw at any time without affecting medical care. Informed consent was obtained electronically via Google Forms (Mountain View, CA: Google LLC). For participants aged 14-17 years, assent from the participant and consent from a parent or legal guardian were obtained.

The study utilized a structured, previously validated questionnaire (WHO Global Adult Tobacco Survey) adapted from published tools assessing tobacco use, risk awareness, and cessation behaviors [16]. The questionnaire was pilot-tested on a small sample of inpatients (n=15) to ensure clarity and cultural appropriateness. The questionnaire consisted of the following four main sections: socio-demographic variables - age, gender, marital status, education level, and occupation. Questions were multiple-choice or categorical; smoking-related variables - type of tobacco used (cigarettes, shisha, or dual use), frequency (average number of cigarettes per day or shisha sessions per week), duration of smoking in years, age at smoking initiation, previous attempts to quit, reasons for smoking (multiple-choice, including stress, social habit, peer influence), and factors contributing to prior quit failures (e.g., withdrawal symptoms, lack of support, relapse triggers); awareness variables - knowledge of health risks associated with smoking (lung cancer, cardiovascular disease, chronic respiratory disease, pregnancy-related outcomes, others) using yes/no or multi-select responses, source of information (physician, media, social networks, family/friends); and cessation-related variables - use of nicotine replacement therapy or pharmacotherapy (e.g., bupropion, varenicline), physician counseling received, intention to quit within the next six months, self-rated motivation to quit (Likert scale: 1-5).

Data were collected electronically by trained co-authors to ensure standardization and minimize duplication. Each co-author was assigned a specific group of patients to reduce bias. Responses were checked daily for accuracy and completeness, and any missing or inconsistent entries were verified with participants. All data were stored securely in password-protected databases accessible only to the investigators, ensuring strict confidentiality.

Data analysis

Data were analyzed using IBM SPSS Statistics version 24. All variables were coded before entry, and quality checks were performed to remove duplicate or incomplete responses. Continuous variables were first tested for normality using the Shapiro-Wilk test. Normally distributed continuous variables were reported as mean±SD, while non-normally distributed variables were summarized as median (IQR). Categorical variables were expressed as frequencies and percentages (n, %).

For inferential analysis, appropriate statistical tests were applied. The chi-square test was used to evaluate associations between categorical variables (gender vs. quit attempts). For continuous variables with two groups (e.g., gender differences in duration of smoking), the independent samples t-test was applied when normality was met, while the Mann-Whitney U test was used for non-normally distributed data. For comparisons across more than two groups (e.g., age groups, marital status, education level), One-way ANOVA was used for normally distributed continuous variables, and the Kruskal-Wallis test for non-normally distributed data. Quit attempts and intention to quit were analyzed using the chi-square test. A p≤0.05 was considered statistically significant.

## Results

In this study, the majority of participants, 101 (40.4%), reported smoking between six and 10 cigarettes per day, followed by 74 (29.6%) who smoked 11-20 cigarettes daily. A smaller proportion (58; 23.2%) consumed only one to five cigarettes, while heavy smoking of more than 20 cigarettes was observed in 17 (6.8%) patients. The mean duration of smoking was 12.84±6.42 years, highlighting long-term tobacco use in this group. The mean age at smoking initiation was 18.62±3.75 years, indicating that most individuals began during late adolescence. In terms of tobacco type, 191 (76.4%) smoked only cigarettes, 34 (13.6%) used shisha, while 25 (10%) reported dual use of both cigarettes and shisha (Table 1).

**Table 1:** Smoking frequency, duration, and type of tobacco among patients (n=250). *Cigarettes smoked per day.

Variables		n (%) or mean±SD
Smoking frequency*	1-5	58 (23.20)
	6-10	101 (40.40)
	11-20	74 (29.60)
	>20	17 (6.80)
Duration of smoking (years)		12.84±6.42
Age at smoking initiation (years)		18.62±3.75
Type of tobacco used	Cigarettes	191 (76.40)
	Shisha	34 (13.60)
	Both (cigarettes and shisha)	25 (10.00)

Most participants demonstrated awareness of smoking-related health risks, with 212 (84.8%) identifying its association with lung cancer. Awareness of cardiovascular complications was reported by 178 (71.2%), while 165 (66%) recognized the link with chronic respiratory diseases. In contrast, fewer respondents, 94 (37.6%), were aware of the adverse impact of smoking on pregnancy outcomes. Notably, 21 (8.4%) patients reported no awareness of smoking-related risks at all (Table 2).

**Table 2:** Awareness of smoking-associated health risks (n=250).

Variables	n (%)
Aware of the link with lung cancer	212 (84.8)
Aware of the link with cardiovascular disease	178 (71.2)
Aware of the link with chronic respiratory disease	165 (66.0)
Aware of the effect on pregnancy outcomes	94 (37.6)
Reported no awareness of risks	21 (8.40)

More than half of the participants (139; 55.6%) reported at least one previous quit attempt, although only 34 (13.6%) were able to maintain abstinence for longer than six months. Among cessation aids (27; 10.8%) reported using nicotine replacement therapy, while 16 (6.4%) had used pharmacological agents, such as bupropion or varenicline. Physician counseling was received by 92 (36.8%) respondents. Regarding future intentions, 118 (47.2%) expressed a willingness to quit within the next six months, whereas 84 (33.6%) stated no intention to quit. An additional 48 (19.2%) remained undecided about their plans (Table 3).

**Table 3:** Smoking cessation practices and intentions (n=250).

Variables	n (%)
Previous quit attempt	139 (55.60)
Successful quit attempt (>6 months)	34 (13.60)
Use of nicotine replacement therapy	27 (10.80)
Use of pharmacotherapy (e.g., bupropion, varenicline)	16 (6.40)
Received physician counseling	92 (36.80)
Intention to quit within next 6 months	118 (47.20)
No intention to quit	84 (33.60)
Undecided	48 (19.20)

The majority of participants were male (197; 78.8%), while females accounted for 53 (21.2%). In terms of age distribution, 41 (16.4%) were between 14 and 24 years of age, 76 (30.4%) were 25-34 years, 63 (25.2%) were 35-44 years, and 70 (28.0%) were aged 45 years or above. More than half of the respondents were married (135; 54.0%), whereas 83 (33.2%) were single, and 32 (12.8%) were divorced or widowed. Regarding education, 37 (14.8%) participants had no formal schooling, 88 (35.2%) had primary or secondary education, 73 (29.2%) completed higher secondary education, and 52 (20.8%) had attained a university degree or higher qualification (Table 4).

**Table 4:** Socio-demographic characteristics of patients (n=250).

Variables	n (%)
Gender	
Male	197 (78.80)
Female	53 (21.20)
Age group (years)	
14-24	41 (16.40)
25-34	76 (30.40)
35-44	63 (25.20)
≥45	70 (28.00)
Marital status	
Single	83 (33.20)
Married	135 (54.00)
Divorced/widowed	32 (12.80)
Education level	
No formal education	37 (14.80)
Primary/secondary	88 (35.20)
Higher secondary	73 (29.20)
University or above	52 (20.80)

In the present study, significant differences in smoking behavior were observed across demographic groups. Among genders, males reported a median cigarette consumption of 10 (IQR: 7-15) compared to females with 8 (IQR: 5-12), while the mean duration of smoking was longer in males (13.5±6.7 years) than in females (10.2±5.4 years). Quit attempts were reported by 111 (56.3%) males and 28 (52.8%) females, whereas the intention to quit was noted among 92 (46.7%) males and 26 (49.1%) females, showing a statistically significant association (p=0.021).

Age groups also showed significant variation (p=0.034). Among participants aged 14-24 years, the median cigarettes/day was 6 (4-10), with a mean duration of 4.5±2.3 years; 18 (43.9%) participants made quit attempts, and 23 (56.1%) reported an intention to quit. In the 25-34 years group, median use was 9 (6-13), duration 9.8±4.8 years, quit attempts 40 (52.6%), and intention to quit 40 (52.6%). Among those aged 35-44 years, cigarette use was 11 (8-16) cigarettes per day, with a duration of 14.3±6.1 years; 46 (73.0%) participants attempted to quit, and 29 (46.0%) intended to quit. Participants aged ≥45 years reported the highest consumption of 12 (9-18) cigarettes/day and a duration of 18.1±7.0 years; 35 (50.0%) reported quit attempts and 26 (37.1%) intended to quit.

A significant association was also observed with marital status (p=0.013). Single individuals smoked 8 (5-12) cigarettes/day for 6.7±3.3 years, with quit attempts in 42 (50.6%) and intention to quit in 47 (56.6%). Married participants smoked 11 (8-15) cigarettes/day for 15.3±6.3 years, with 78 (57.8%) attempting to quit and 54 (40.0%) intending to quit. Among divorced/widowed participants, cigarette use was 10 (7-14), duration 13.0±5.7 years, quit attempts 19 (59.4%), and intention to quit 17 (53.1%).

Education level also demonstrated statistically significant differences (p=0.033). Participants with no formal education smoked a median of 12 (IQR: 8-17) cigarettes per day for a mean duration of 15.9±6.9 years; 16 (43.2%) had attempted to quit, and 11 (29.7%) intended to quit. Participants with primary to secondary education smoked 10 (7-14) cigarettes/day for 13.7±6.4 years, with 44 (50.0%) quitting and 41 (46.6%) intending to quit. In the higher secondary group (n=79), cigarette use was 9 (6-12) cigarettes per day, with a duration of 11.2±5.3 years; 41 (56.2%) attempted to quit, and 37 (50.7%) intended to quit. University-educated participants reported the lowest cigarette use, 7 (5-10), and the shortest duration, 8.3±4.2 years, with the highest quit attempts, 38 (73.1%), and intention to quit, 29 (55.8%) (Table 5).

**Table 5:** Smoking behavior stratified by gender, age, marital status, and education (n=250). Cigarettes smoked per day (non-normally distributed) were compared using the Mann-Whitney U test for two-group comparisons and the Kruskal-Wallis test for multiple-group comparisons. Duration of smoking (normally distributed) was analyzed using the independent samples t-test and one-way ANOVA. Quit attempts and intention to quit were analyzed using the chi-square test. A p≤0.05 was considered statistically significant.

Category	Group	Cigarettes/day (median {IQR})	Duration (years, mean±SD)	Quit attempt, n (%)	Intention to quit, n (%)	p-Value
Gender	Male (n=197)	10 (7-15)	13.5±6.7	111 (56.3)	92 (46.7)	0.021
	Female (n=53)	8 (5-12)	10.2±5.4	28 (52.8)	26 (49.1)	
Age (years)	14-24 (n=41)	6 (4-10)	4.5±2.3	18 (43.9)	23 (56.1)	0.034
	25-34 (n=76)	9 (6-13)	9.8±4.8	40 (52.6)	40 (52.6)	
	35-44 (n=63)	11 (8-16)	14.3±6.1	46 (73.0)	29 (46.0)	
	≥45 (n=70)	12 (9-18)	18.1±7.0	35 (50.0)	26 (37.1)	
Marital status	Single (n=83)	8 (5-12)	6.7±3.3	42 (50.6)	47 (56.6)	0.013
	Married (n=135)	11 (8-15)	15.3±6.3	78 (57.8)	54 (40.0)	
	Divorced/widowed (n=32)	10 (7-14)	13.0±5.7	19 (59.4)	17 (53.1)	
Education	None (n=37)	12 (8-17)	15.9±6.9	16 (43.2)	11 (29.7)	0.033
	Primary/secondary (n=88)	10 (7-14)	13.7±6.4	44 (50.0)	41 (46.6)	
	Higher secondary (n=79)	9 (6-12)	11.2±5.3	41 (56.2)	37 (50.7)	
	University or above (n=46)	7 (5-10)	8.3±4.2	38 (73.1)	29 (55.8)	

## Discussion

This cross-sectional survey found that most inpatients smoked at moderate daily levels, had long cumulative exposure (mean duration: 12.84±6.42 years), and typically initiated smoking in late adolescence (mean age: 18.62±3.75). The predominance of cigarette smoking over shisha and dual use in our sample aligns with reports from Saudi clinical and student populations showing cigarettes remain the most common tobacco product, though patterns vary by subgroup [17,18]. Recent national data suggest that adult smoking prevalence in Saudi Arabia has remained stable or increased slightly, indicating that the inpatient trends observed here reflect broader population patterns.

Awareness of major smoking harms was high for lung cancer (212; 84.8%) and cardiovascular disease (178; 71.2%), but substantially lower for pregnancy outcomes (94; 37.6%). High general awareness of cancer and cardiac risks has been observed in other Saudi studies, while knowledge of reproductive harms is often weaker, particularly among younger and less-educated groups, a pattern consistent with national surveys and university samples [19,20]. Sources of participants’ knowledge, physicians, media, peers, and family, were recorded, highlighting potential avenues for targeted education.

Over half of participants reported at least one prior quit attempt (139; 55.6%), but sustained abstinence (>6 months) was uncommon (34; 13.6%). Use of pharmacological supports (16; 6.4%) and nicotine replacement therapy (27; 10.8%) was low, and only 92 (36.8%) reported having received physician counseling. These figures echo national observations that quit attempts are frequent but successful long-term cessation remains limited, and utilization of formal cessation services and pharmacotherapy is low. The modest reach of cessation clinics and variability in counseling quality may partly explain these low uptake and success rates [21]. Although emerging nicotine products such as e-cigarettes and vaping are increasingly adopted by Saudi youth and adults, these were not assessed in the current study, which may underestimate the overall nicotine use burden.

Stratified analyses showed statistically significant socio-demographic gradients as follows: males smoked more intensively and for longer periods than females, smoking frequency and duration increased with age, married participants had higher frequency and duration than singles, and lower educational attainment was associated with heavier, longer smoking and fewer intentions/attempts to quit. The strong male predominance and gender differences in intensity are consistent with prior Saudi studies reporting higher male prevalence and heavier consumption among men [22,23]. Age-related accumulation of duration and higher nicotine exposure in older cohorts has been described in both community and hospital samples and reflects cohort effects and cumulative exposure [18,24]. The inverse relationship between education and smoking intensity/duration in our sample, together with greater quit attempts and intentions among higher-educated patients, supports the socio-economic gradient frequently reported in tobacco epidemiology [25,26].

Nearly half of patients expressed intention to quit within six months (118; 47.2%), but far fewer had used evidence-based aids, highlighting an implementation gap as follows: motivation exists, but access to or uptake of effective interventions is limited. This mirrors evidence from Saudi evaluations of cessation services, which show reasonable awareness but low utilization of fixed clinics and underuse of pharmacotherapy, suggesting an opportunity for stronger hospital-based cessation delivery and linkage to national services [19,21,27]. Compared with studies of university and young adult populations in Jeddah, our inpatient sample showed an older mean smoking duration and more intense cumulative exposure, as expected for a hospitalized cohort with a higher comorbidity burden. However, the early age of initiation in our study is consistent with reports that many Saudi smokers begin in adolescence or early adulthood, indicating the need for continued primary prevention targeting teens and young adults [24,28,29].

Clinical and public-health implications of these results are threefold. First, tertiary-care hospitals are important touchpoints for cessation interventions, given the high prevalence of long-term smokers among admitted patients and their expressed readiness to quit. Second, the low use of pharmacotherapies and limited physician counseling point to missed opportunities for brief advice, prescription of cessation medications, and structured referral to smoking cessation clinics. Strengthening in-hospital cessation pathways, including training clinicians, implementing opt-out brief interventions, and offering nicotine replacement therapy (NRT)/pharmacotherapy during admission, could improve quit rates. Third, targeted education is needed to fill specific knowledge gaps (e.g., effects on pregnancy), which could be integrated into discharge counseling, community outreach, and aligned with Saudi Vision 2030 Health Sector Transformation Program and hospital accreditation standards that require cessation services [30].

This study has several limitations. The cross-sectional design precludes causal inference and may be subject to recall and social desirability bias, particularly for self-reported quitting history and intentions. The single-center inpatient sample limits the generalizability of the findings to community populations or other regions. Pharmacotherapy and counseling utilization were self-reported and not verified through prescription records or cessation clinic documentation. The study focused on traditional tobacco products (cigarettes and shisha) and did not assess e-cigarettes or other emerging nicotine delivery systems, which may have led to an underestimation of nicotine use. Finally, some subgroup cell sizes (e.g., female smokers) were relatively small, which may affect the precision of stratified estimates.

## Conclusions

This cross-sectional survey highlights that smoking remains a significant health concern among hospitalized patients in Jeddah, characterized by high daily consumption, early initiation, and notable gaps in awareness of specific health risks. Although many participants had previously attempted to quit, sustained cessation was uncommon, and the use of evidence-based supports, including nicotine replacement therapy and pharmacotherapy, was limited. Pronounced socio-demographic disparities, particularly higher smoking intensity among males, older individuals, married patients, and those with lower educational attainment, underscore the need for tailored, hospital-based cessation strategies. Strengthening inpatient cessation pathways through systematic screening, clinician-led counseling, and provision of pharmacological interventions can help capitalize on hospitalization as a critical window to reduce the burden of smoking-related diseases and support long-term behavior change. Future studies should also assess emerging nicotine delivery systems, such as e-cigarettes and vaping, and link findings to national tobacco-control objectives to inform more comprehensive hospital- and policy-level cessation strategies.
